# Cognitive Outcome Prediction in Infants With Neonatal Hypoxic-Ischemic Encephalopathy Based on Functional Connectivity and Complexity of the Electroencephalography Signal

**DOI:** 10.3389/fnhum.2021.795006

**Published:** 2022-01-27

**Authors:** Noura Alotaibi, Dalal Bakheet, Daniel Konn, Brigitte Vollmer, Koushik Maharatna

**Affiliations:** ^1^School of Electronics and Computer Science, University of Southampton, Southampton, United Kingdom; ^2^Department of Computer Science and Artificial Intelligence, University of Jeddah, Jeddah, Saudi Arabia; ^3^Clinical Neurophysiology, University Hospital Southampton, Southampton, United Kingdom; ^4^Clinical Neurosciences, Clinical and Experimental Sciences, Faculty of Medicine, University of Southampton, Southampton, United Kingdom; ^5^Paediatric Neurology, Southampton Children’s Hospital, Southampton, United Kingdom

**Keywords:** brain connectivity, cognitive scores, graph theory, electroencephalography (EEG), entropy analysis, hypoxic-ischemic encephalopathy (HIE), noise-assisted multivariate empirical mode decomposition (NA-MEMD)

## Abstract

Impaired neurodevelopmental outcome, in particular cognitive impairment, after neonatal hypoxic-ischemic encephalopathy is a major concern for parents, clinicians, and society. This study aims to investigate the potential benefits of using advanced quantitative electroencephalography analysis (qEEG) for early prediction of cognitive outcomes, assessed here at 2 years of age. EEG data were recorded within the first week after birth from a cohort of twenty infants with neonatal hypoxic-ischemic encephalopathy (HIE). A proposed regression framework was based on two different sets of features, namely graph-theoretical features derived from the weighted phase-lag index (WPLI) and entropies metrics represented by sample entropy (SampEn), permutation entropy (PEn), and spectral entropy (SpEn). Both sets of features were calculated within the noise-assisted multivariate empirical mode decomposition (NA-MEMD) domain. Correlation analysis showed a significant association in the delta band between the proposed features, graph attributes (radius, transitivity, global efficiency, and characteristic path length) and entropy features (Pen and SpEn) from the neonatal EEG data and the cognitive development at age two years. These features were used to train and test the tree ensemble (boosted and bagged) regression models. The highest prediction performance was reached to 14.27 root mean square error (RMSE), 12.07 mean absolute error (MAE), and 0.45 *R*-squared using the entropy features with a boosted tree regression model. Thus, the results demonstrate that the proposed qEEG features show the state of brain function at an early stage; hence, they could serve as predictive biomarkers of later cognitive impairment, which could facilitate identifying those who might benefit from early targeted intervention.

## Introduction

Hypoxic-ischemic encephalopathy (HIE) is one of the most severe birth complications that causes neonatal brain damage. The incidence of HIE is approximately 1–6 per 1000 live births (Byeon et al., 2015). Moderate to severe encephalopathy often leads to death, cerebral palsy, or severe neurodevelopmental impairment. Neurodevelopmental impairment (NDI) is a composite outcome that includes cognitive, behavioral, educational, and motor impairments. Cognitive deficit is considered one of the most expected outcomes associated with NDI, featured by slow information processing speed, deficits in working memory, attention, and executive function (Slaughter et al., 2016). This substantially impacts the affected individual and their families, including education, social participation, employment, and quality of life.

Early identification of infants at high-risk can help to provide targeted early intervention that aims to improve cognitive outcomes by taking advantage of the neuroplasticity of the developing brain in early infancy.

Recently, there has been increasing interest in exploring methods for assessing brain function in early infancy and using them as an aiding tool for the early prediction of cognitive impairments. Neuroimaging techniques have been used in several studies to identify infants at high-risk of cognitive impairment (Slaughter et al., 2016; Moeskops et al., 2017; He et al., 2018). Alongside neuroimaging methods, electroencephalography (EEG) is suggested to be the current gold standard technique for studying brain activity as it is relatively inexpensive, portable, non-invasive, user-friendly, and comparatively easy to use. Several studies have examined the feasibility of using EEG analysis to predict the cognitive outcome. Kong et al. (2018) conducted a systematic review highlighting the two basic approaches currently adopted for the early prediction of cognitive outcomes. One is the analysis of EEG features to identify the biomarkers that could help binary classify the subject as either cognitively impaired or normal. The second is the analysis of EEG characteristics to estimate the specific scores for a continuous cognitive measure that could predict cognitive performance. Compared to binary classification, prediction of cognitive development reflects the difference between individuals in terms of brain function and the levels of cognitive impairment, rather than determining the group membership as in the case of classification, which could be more challenging (Sui et al., 2020).

Limited previous studies have shown that early quantitative analysis of EEG can satisfactorily predict long-term cognitive outcome. Lloyd et al. (2021) employed serial, multichannel video EEG to predict outcome in preterm infants by finding the association between grading of EEG background activity–where EEG was recorded soon after birth and continued over the first 3 days–and developmental scores, at 2 years of age. Suppiej et al. (2017) compared spectral EEG values of infants born near term with infants born at extremely low gestational age, aiming to investigate whether spectral EEG features were related to neurological outcomes. The EEG data was recorded at 35 weeks post-conception, and the outcome was evaluated at 1 year of age with the Griffiths’ scales. Cainelli et al. (2021) carried out a longitudinal 6-year study to evaluate the feasibility of neonatal spectral EEG in predicting developmental delay in premature infants. The EEG data was recorded at 35 weeks post-conception, and the outcome was assessed at 6 years using Wechsler Pre-school and Primary Scale of Intelligence III. West et al. (2005) conducted regression-based analysis to predict outcomes at 18 months of 44 preterm infants using the quantitative measure of EEG continuity recording in the first 4 days after birth. Kühn-Popp et al. (2016) investigated the relationship between brain maturation processes and language skills (evaluated at 48 months) using EEG coherence measured at 14 months.

Although these attempts have paved the way to using EEG in early prediction of cognitive development, methodological limitations hinder further progress. For example, EEG grading systems are still subjective and dependent on interpretation by an expert.

On the other hand, coherence-based measures quantitatively estimate the linear similarity of relative amplitude and phase between signals in a specific frequency range. Although coherence analysis provides information on the degree of synchrony of brain activity at different locations for each frequency, it suffers from several limitations. First, it fails to capture the intrinsic non-linearity of brain activity, is unsuitable for tracking non-stationary dynamics as it partly depends on the amplitudes of the signals and is susceptible to the volume conduction issue (Sweeney-Reed and Nasuto, 2007). Moreover, coherence relies on both the amplitude and phase in its calculation, and there is increasing evidence that considering the synchronization of phase alone and separating it from amplitude information may allow capturing the synchrony of temporal information between signals. This temporal locking of phases between neural signals is considered essential for analyzing the dynamic neural assemblies underlying cognitive processing (Sweeney-Reed and Nasuto, 2007).

Spectral power can quantitatively capture EEG characteristics that could objectively predict the associated cognitive outcomes. However, the employed approaches are based on Fourier transform, which requires linearity and stationarity of the signals, which is not the case with EEG signals. Consequently, such spectral analysis methods may give misleading amplitude-frequency distribution for non-linear and non-stationary data. In addition, the spectral analysis methods used in the literature are based on *a priori* basis, often selected according to traditional frequency bands, which are inconsistent among studies. For example, alpha-band was chosen to be from 8 to 12 Hz in David et al. (2004), from 8 to 13 Hz in Breakspear et al. (2004), from 8 to 14 in Babiloni et al. (2006), or subdivided into 6–10 Hz and 10–14 Hz ranges in Stam et al. (2003). These small changes in the frequency ranges of interest may result in potentially informative brainwaves being missed, specifically in the case of infants, due to the well-known variability between them and the older individuals in the neural oscillations of interest (Saby and Marshall, 2012). Furthermore, using *a priori* basis is critical for both non-linear and non-stationary data, as one cannot expect a predetermined basis to fit all the non-linear and non-stationary dynamics (Huang et al., 1998).

Thus, further work is required to employ quantification and analysis methods that consider the non-linearity and non-stationary characteristics of EEG, intending to find objective and reliable biomarkers of the cognitive deficits.

This study, therefore, aims to investigate the effectiveness of non-linear quantitative EEG (qEEG) features within the regression-based framework for predicting the cognitive outcomes in term-born infants with neonatal HIE. Specifically, phase-based functional brain connectivity estimated by weighted phase-lag index (WPLI) with graph metrics and complexity analysis measured by sample entropy (SampEn), permutation entropy (PEn), and spectral entropy (SpEn) are the two classes of features used in this study. Both sets of features were previously validated on earlier prediction of CP in at high-risk infants with neonatal HIE (Bakheet et al., 2021). This study uses WPLI and entropies features to find the association between neonatal EEG and the individual cognitive performance (which were completed in a follow-up visit at 24 months of age). These features are chosen because both could capture the complex characteristics of the EEG spectra, particularly non-linear and non-stationary properties. While WPLI quantifies the phase synchronization between distinct brain areas over time, providing a global view of the whole-brain networks, the entropies measure the complexity of each EEG independently, providing an understanding of the dynamic process underlying specific brain area.

In order to estimate such features, it is usually necessary to decompose EEG signals into narrowband components. This step is required since the calculation of WPLI and entropies from a complex signal, as in EEG, composed of multiple frequency oscillators, does not reveal the underlying non-linear dynamics of the signals (Takahashi et al., 2010; Bruña et al., 2018). Thus, noise-assisted multivariate empirical mode decomposition (NA-MEMD) method is adopted to decompose the EEG signals into intrinsic components. One advantage of using NA-MEMD is that it is a fully adaptive method and does not require *a priori* selection of the filter cut-offs. Naturally, this is a valuable property because it can tackle the well-known frequency range variability according to the experimental condition. Another advantage of using NA-MEMD is it capable of dealing with the non-stationary property of EEG signals by demonstrating the temporal evolution of different frequency components.

Correlation analysis is performed to ascertain the statistical significance of graph-theoretical parameters of WPLI and entropies features in finding the association with cognitive scores. Then, the significant features are used to train and test the tree ensemble regression models: boosting and bagging to evaluate their predictability in later cognitive development.

The remainder of the article is organized as follows: the materials and methods used in this study are described in section “Materials and Methods.” Results are analyzed in section “Results” and discussed in detail in section “Discussion.” Section “Conclusion” concludes the article.

## Materials and Methods

**FIGURE 1:**
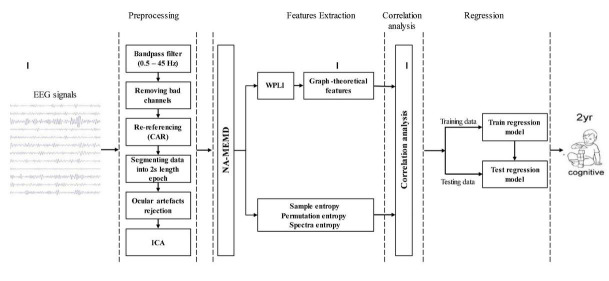
Schematic outline of proposed analysis for predicting cognitive outcomes at 2 years of age.

This section describes the methodology of the proposed analysis to predict cognitive development at 2 years of age. First, a description of the study population and recruitment process is given, followed by a description of the experimental setup. An overview of the overall process of the proposed analysis including the description of pre-processing techniques, the basic concept of NA-MEMD, WPLI-based functional brain connectivity analysis, graph theory, and complexity analysis, are also provided. Then, the procedure of how these methods is employed to extract the desired features will be demonstrated. Finally, the regression models used in this study are presented. A schematic outline of the proposed analysis is depicted in Figure 1.

### Participants

Thirty term-born infants with HIE treated with hypothermia were recruited in this study. EEG data was recorded on the neonatal intensive care unit within the first week after birth. Routine clinical neurodevelopmental follow-up assessment was conducted at 24 months of age using the Bayley Scales of Infant and Toddler Development III (BSITD-III). Of the 30 infants, 20 completed the follow-up assessment. The BSITD-III consists of three scales; motor, language and cognitive, and for this study we used the composite scores from the cognitive scales. The BSITD-III cognitive scores from those twenty infants ranged from 74 to 145. The BSITD-III mean of the normal population is 100, with a standard deviation (SD) of 1.5. Delay of cognitive development was defined as cognitive scores > 1.5 SD below the norm population mean. Ethical approval for secondary analysis of anonymized clinical data was obtained by the HRA and Health and Care Research Wales, HCRW (Reference ID 20/HRA/0260; IRAS project ID 278072; University Hospital Southampton R&D protocol number RHM CHI1047).

### Data Acquisition

**FIGURE 2:**
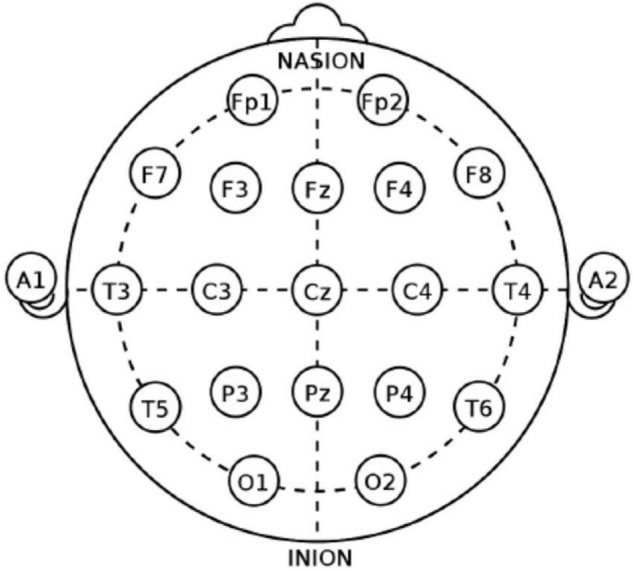
The 10–20 international system of EEG electrodes placement.

Electroencephalography data was recorded for 20 min during resting-state condition with eyes closed by either a Nihon Kohden (sampling frequency 1000 Hz, high-pass filter 0.016 Hz, the low-pass filter 300 Hz) and XLTEK (sampling frequency 512 Hz, high-pass filter 0.1 Hz, and the low-pass filter 70 Hz) clinical video-EEG system. Nineteen electrodes (C3, C4, CZ, F3, F4, F7, F8, FZ, FP1, FP2, O1, O2, P3, P4, PZ, T3, T4, T5, and T6) placed according to the 10–20 international system were used, as shown in Figure 2. Movement or electrode artifact affected the EEG in a substantial proportion of the cases. The first period in the EEG that was long enough without any clear significant artifact was always selected (the average length of the clips is approximately 2 min).

### Data Pre-processing

The EEG data analysis was performed using MATLAB software package R2018a and EEGLAB toolbox. In order to improve the quality of the EEG signal, the remaining artifacts such as eye movement, muscle, heart activities, line noise, and signal discontinuity were eliminated using the following pre-processing techniques.

The EEG signals were initially recorded from 19 channels where the standard ground electrode was put close to Fz or Cz. After filtering the data, the EEG signals were automatically inspected to determine the set of bad channels. The procedure of picking the bad channels is based on the two criteria: first, the flat channels, and second, the channels with a large amount of noise determined based on their standard deviation. Any channel marked as bad was eliminated and excluded from the further analysis. As a result of bad channels detection, the total number of removing channels were seven: C4, CZ, F4, F8, FP1, FP2, and Pz. The set of remaining channels used in calculating the common averaged reference (CAR) were 12: C3, F3, F7, Fz, O1, O2, P3, P4, T3, T4, T5, and T6. Thus, the brain topography used in this study included three brain regions: the central (C3, T3, and T4), anterior (F3, F7 and Fz), and posterior (O1, O2, P3, P4, T5, and T6) lobes.

A CAR was applied to re-reference the data to mitigate the confounding effect of the reference. According to a typical approach of EEG resting-state analysis, the EEG signal recorded from each channel was divided into epochs (windows), each of 2 s duration (Kułak and Sobaniec, 2003). This was chosen according to the natural properties of EEG, which are non-stationary in general and, however, it is quasi-stationary within only a short interval (Sakkalis, 2011). Thus, 2 s length appears to be the most appropriate length for capturing such EEG characteristics.

Epochs contaminated with ocular artifacts, particularly eye movement, were automatically identified using EEGLAB. A certain threshold of 55 μV was set and applied on each window; any epoch above the threshold was rejected and not used in further analysis (Apicella et al., 2013). Independent component analysis (ICA) was then applied, using the runICA algorithm implemented in EEGLAB, to remove the remaining artifacts from the signals, such as muscle artifacts and cardiac activity. Thus, the EEG signals from the 12 channels were separated into their 12 constituent independent components (ICs), as the general rule of ICA is to find the *N* ICs from the *N* linearly mixed signal (input channel data). These ICs are then projected back to the EEGs using the estimated separating matrix after eliminating the artifact-related ICs according to Chaumon et al. (2015). The algorithm of ICA was briefly described as follow:

The ICA decomposition finds an unmixing matrix (*W*) that decomposes the input channel data (x) into a sum of temporally independent and spatially fixed components, *u* = Wx. The rows of this output data matrix, u, called the component activations, are time courses of activation of the ICA components, and the columns of the inverse matrix *W*^−1^ give the projection strengths of the respective components onto the scalp sensors. The scalp topographies of the components provide information about the location of the sources (e.g., eye activity should project mainly to frontal sites, etc.). The classification of these components as artifact or EEG signal was performed using visual inspection based on the scalp topographies of the component. The artifact-free EEG data *x*′ was fully reconstructed by multiplying of the inverse of W with *u*′, where *u*′ is the matrix of component activation, u, with rows representing artefactual components set to zero.


(1) $$x^{\prime }=W^{-1}\,u^{\prime }$$

The remaining epochs after the rejecting process were slightly varied between subjects. Since the infant who yields the lowest number of epochs upon rejection process gives 30 epochs, the first 30 epochs of each infant were considered. Thus, for each infant, a total of 12 channels, each with 30 artifact-free of 2 s EEG epochs, were used in the next stage of analysis.

### Noise-Assisted Multivariate Empirical Mode Decomposition

Noise-assisted multivariate empirical mode decomposition is a data-driven time-frequency analysis capable to deal with non-stationary data (ur Rehman and Mandic, 2011). It was employed in this study to decompose the EEG signals into finite oscillation scales at the time domain. The proposed set of features was then calculated from each scale to characterize the time-frequency integration of information.

Noise-assisted multivariate empirical mode decomposition is a modified extension of empirical mode decomposition (EMD), figuring out its mode-alignment and mode-mixing problems (Huang et al., 1998). The mode-alignment refers to the issue of getting non-identical numbers of components when decomposing a multivariate signal, while the mode-mixing points out the situation of having different frequency ranges in a single scale. NA-MEMD solves the mode-mixing problem by adding a subspace containing multivariate independent white noise to the original multivariate signal, and then it processes the resulting composite signal using the multivariant empirical mode decomposition (MEMD) algorithm, which was proposed earlier to settle the mode-alignment (ur Rehman et al., 2010).

Unlike other traditional decomposing methods such as short-time Fourier (Gabor, 1946), wavelet transform (Mallat, 1989) and band-pass filters, EMD-based methods do not require a predefined basis of the signals. Instead, they decompose the time-series adaptively, through the Sifting process, from high to low-frequency components known as intrinsic mode functions (IMFs).

Among the available decomposition methods, the well-established wavelet analysis is known as one of the best non-stationary data analysis methods (Huang et al., 1998). However, the predefined basis of, for example, the Morlet wavelet (the most commonly used wavelet in general and in EEG analysis) leads to different issues (Sweeney-Reed and Nasuto, 2007). First, one cannot guarantee that the predetermined window size of the wavelet will coincide with a stationary period. Good localisation of the low-frequency oscillations needs a long-time window to identify them and thus a longer period of time for which signal should be stationary. On the other hand, selecting a small window may lead to missing potential biomarkers in the lower frequency ranges. Such a situation is known as the uncertainty principle, produced from the trade-off between frequency and time. Second, the prior selection of wavelet parameters cannot be expected to fit all the non-linear and non-stationary phenomena. Thus, it could induce spurious harmonic components to spectrally represent the signals, causing energy spreading and leading to faulty results.

Empirical mode decomposition-based methods satisfy the necessary conditions for the decomposition to represent a non-linear and non-stationary time series, particularly locality and adaptivity conditions. The locality is most crucial for non-stationarity, in which all events have to be identified by the time of their occurrences. Thus, both the amplitude (or energy) and the frequency are required to be functions of time (Huang et al., 1998). The adaptivity is important for both non-linear and non-stationary data in which the decomposition is adapted to the local variations of the data and hence can fully account for the underlying dynamics of the signals (Huang et al., 1998). Different studies proved that the local and adaptive nature of the decomposition using EMD-based methods is shown to improve time and frequency precision compared to the Morlet wavelet (Huang et al., 1998; Sweeney-Reed and Nasuto, 2007). A comparative summary of the Morlet wavelet and EMD-based methods is given in Table 1.

**TABLE 1:** A comparative summary of the Morlet wavelet and EMD-based methods.

	Morlet wavelet	EMD-based methods
Basis	*a priori*	adaptive
Time-frequency precision	uncertainty	certainty
Non-linear	no	yes
Non-stationary	yes	yes
Theoretical base	yes	no (empirical)

The procedure of the sifting process of the NA-MEMD method starts by considering a sequence of *n*-dimensional vectors ${\left\{v\left(t\right)\right\}}_{t=1}^{T}=\left\{v_{1}\left(t\right),v_{2}\left(t\right),v_{3}\left(t\right),\ldots ..,v_{n}\left(t\right)\right\}$ that represents a multivariate signal with *n* components (including the original signals and the added noise), and a set of direction vectors $X^{Q_{k}}=\left\{x_{1}^{k},x_{2}^{k},x_{3}^{k},\ldots \,\ldots ,x_{n}^{k}\right\}$ along the directions given by angles $Q^{k}=\left\{Q_{1}^{k},Q_{2}^{k},\ldots ,Q_{\left(n-1\right)}^{k}\right\}$ on an (*n*−1)-sphere. Then the MEMD algorithm is applied as follows:

1) Choose a suitable set of points for sampling on a (*n*−1) sphere.
2) Calculate a projection, denoted by${\left\{P^{Q_{k}}\,\left(t\right)\right\}}_{t=1}^{T}$, of the input signal ${\left\{v\,\left(t\right)\right\}}_{t=1}^{T}$along the direction vector*X^Q^*_*k*_, for all *k* (the whole set of direction vectors), giving ${\left\{P^{Q_{k}}\,\left(t\right)\right\}}_{k=1}^{K}$as the set of projections.
3) Find the time instants $t_{j}^{Q_{k}}$ corresponding to the maxima of the set of projected signals${\left\{P^{Q_{k}}\,\left(t\right)\right\}}_{k=1}^{K}$.
4) Interpolate $\left[t_{j}^{Q_{k}},v\,\left(t_{j}^{Q_{k}}\right)\right]$ to get the multivariate envelope curves${\left\{e^{Q_{k}}\,\left(t\right)\right\}}_{k=1}^{K}$.
5) For a set of *K* direction vectors, the mean *m*(*t*) of the envelope curves is calculated as $m\,\left(t\right)=\frac{1}{K}\,\sum_{k=1}^{K}e^{Q_{k}}\,\left(t\right)$.
6) Extract the detail *c*_*i*_(*t*) using *c*_*i*_(*t*) = *v*(*t*)−*m*(*t*) (*i* is an order of IMF). If the detail *c*_*i*_(*t*) satisfies the IMF conditions, apply the above procedure to *v*(*t*)−*c*_*i*_(*t*), otherwise apply it to *c*_*i*_(*t*).

The sifting process can be stopped when the detail *c*_*i*_(*t*) is monotonic and no more IMFs can be extracted from it.

### Weighted Phase-Lag Index-Based Functional Brain Connectivity Analysis

Functional brain connectivity is an established technique for getting insight into the process of information propagation and relationship strength amongst the brain areas–the underpinning mechanism of the working principle of the brain. WPLI is the non-linear measure of functional brain connectivity that quantifies the phase synchronization between two signals (Vinck et al., 2011). It is an extended version of the phase-lag index (PLI) providing a better estimation of connectivity than PLI that is hindered by the discontinuity issue (Stam et al., 2007). This problem occurs in the case of small phase perturbation that could turn the phase lags into leads and vice versa (Vinck et al., 2011). WPLI was proposed to alleviate the effect of discontinuity of the connectivity index, volume conduction and other sorts of noise. It gives a reliable estimation of connectivity because it considers the magnitudes of the imaginary component of the cross-spectrum for weighting the phase differences between two sources of signals. Accordingly, the phase differences at high-risk for changing their true signs under the effect of small noise perturbations are assigned to small weight equivalent to the magnitude of the imaginary component. Subsequently, they would have a lower impact in quantifying connectivity.

Mathematically, WPLI can be defined as:


(2) $$W\,P\,L\,I=\frac{\left|\left\langle |ℑ\left(X\right)|\text{sign}\left(ℑ\left(X\right)\right)\right\rangle \right|}{\left\langle \left|ℑ\,\left(X\right)\right|\right\rangle }$$

where ℑ(*X*)| is the imaginary component of the cross-spectrum *X* for two real-valued signals *Z*_1_ and *Z*_2_. The cross-spectrum *X* is computed as:


(3) $$X=Z_{1}*Z_{2}^{*}$$

where $Z_{2}^{*}$ is a complex conjugate of *Z*_2_. The value of WPLI ranges between 0 and 1, with 0 referring to no coupling between two signals, whereas one indicates that the two signals are perfectly coupled. WPLI quantified the functional brain connectivity between all twelve channels. Since a phase estimation would be better if it was extracted from a narrow frequency range in each source, in this study, the NA-MEMD method was adopted to decompose EEG signals into the intrinsic components. Then, these components were subjected to instantaneous phase estimation.

### Fundamental Graph Theory

Graph theory is often applied to functional brain connectivity to describe the network architecture (Vecchio et al., 2017). In the graph theory concept, the brain can be represented as a network where the nodes correspond to distinct brain regions or EEG electrodes in EEG-based functional brain connectivity derivation, and the edges representing the functional connections between them. It can adequately characterize the network’s topology and provide quantitative information about its properties. The graph-theoretical parameters measure these topological properties on both local and global scale. Local attributes identify the topological features of the single node, while the global metrics reveal the information flow over the whole network as well as any specialized local processing. There is increasing evidence that pathological conditions are viewed as a dynamic process affecting the entire brain.

On this basis, neuroimaging results have suggested that applying global attributes to quantify the global network topological properties helps to reveal the disruptions in brain network behind such pathological conditions. Accordingly, in this study, the global parameters were chosen to investigate the whole topological properties of an infant’s brain network. Identifying an aberrant in this network characteristics is expected to show the potential cognitive deficit emerging later during the child’s lifespan. Mainly, transitivity, global efficiency, radius, diameter, and characteristic path length were used for this purpose. Global efficiency and characteristic path length are the measures of network integration referring to the ability of the network to transfer the information concurrently over the network. Radius and diameter provide insight into network eccentricity, while transitivity quantifies the ability of the network to localize information processing responsible for specific functions (Zhao et al., 2019). From the information processing perspective, networks possessing a high global efficiency and short characteristic path length have high efficiency in global information transfer and a high degree of network integration. On the other hand, networks with either a low radius or diameter have a high ability of information integration between brain areas. On another hand, the networks possessing high transitivity have a high local information transfer and these networks have a high tendency to specialize processing certain functions within a highly interconnected sub-network (Zhao et al., 2019). Even though the modularity measures the strength of the tendency of the network to divide into modules or groups, it is not considered in this study. This is because it can explain the capacity of a network of processing the local information rather than providing a view about the global information transfer within the network.

Those graph-theoretical parameters were computed using brain connectivity toolbox (BCT) in a MATLAB environment (Mika, 2010), and a brief description is illustrated in Table 2.

**TABLE 2:** List of graph parameters that used for characterizing a functional brain network.

Feature	Description
**Transitivity**	Reflecting connectivity of given region to its neighbors. The network with high transitivity implies it contains groups of regions that are densely connected internally.
**Global efficiency**	Representing the inverse of the shortest path between the regions. It measures the network efficiency in terms of how well the brain network integrated and how easily the information transfer between distinct brain regions.
**Radius**	Measuring the shape of network and it is the minimum of the network eccentricity.
**Diameter**	Measuring the shape of the network and it is the maximum of network eccentricity.
**Characteristic path length**	Representing the average distance between all pairs of brain regions in the network. It indicates how easily information transforms across the network.

### Complexity Analysis

Different studies in the literature report atypical EEG complexity associated with various neurodevelopmental disorders (Takahashi et al., 2010). Complexity analysis is utilized to provide a non-linear estimation of the dynamical brain activity. Entropy-based measures are commonly used to quantify time series complexity (Takahashi et al., 2010). Brief descriptions of the entropy measures employed in this study are given in the following.

#### Sample Entropy

Sample entropy, proposed by Richman and Moorman, provides an estimation of the irregularity or randomness of a time series (Richman and Moorman, 2000). It is a modified version of approximate entropy (ApEn) (Pincus, 1991), improving its immunity to the noise in the data and sensitivity to the signal length (Richman and Moorman, 2000). Both measures have been widely employed for the analysis of physiological data. SampEn measures the probability that two similar patterns for *m* point remain similar at the next *m*+1 point within a tolerance *r*. Thus, for the time series *x(i)* of length *N*, SampEn is given by:


(4) $$S\,a\,m\,p\,E\,n\,\left(m,r,N\right)=-\ln\left[A^{m}\,\left(r\right)/B^{m}\,\left(r\right)\right],$$

where


(5) $$A^{m}\,\left(r\right)={\left(N-m\right)}^{-1}\,\sum_{i=1}^{N-m}C_{i}^{m+1}\,\left(r\right),$$

(6) $$B^{m}\,\left(r\right)={\left(N-m\right)}^{-1}\,\sum_{i=1}^{N-m}C_{i}^{m}\,\left(r\right),$$

(7) $$C_{i}^{m}\left(r\right)={\left(N-m-1\right)}^{-1}C_{i},i=1,2,..,N-m,$$

where *m* is the embedding dimension, *B*^m^*(r)* is the likelihood that *X_*m*_(i)* and *X_*m*_(j)* is matching for *m* points, while *A*^m^*(r)* is the likelihood that *X_*m*_(i)* and *X_*m*_(j)* will match for *m*+1 points. *C_*i*_*^m^*(r)* is the probability of a vector *X_*m*_(i)* being similar to *X_*m*_(j)* within a tolerance *r*, *C*_*i*_ is the number that the distance two vectors *X(i)* and *X(j)* is smaller than *r*, and a vector *X*_*m*_(*i*)(1≤*i*≤*N*−*m* + 1) reconstituted of this series, and is given by: *X*_*m*_(*i*) = {*x*(*i*),*x*(*i* + 1),…,*x*(*i* + *m*−1)}. For optimal estimation of SampEn, some studies have recommended the embedding dimension *m* = 2 or 3, and the tolerance *r* = 0.1–0.25 of the standard deviation of the signal (Bruhn et al., 2000; Tudor et al., 2005). In this investigation, different parameter settings in these recommended ranges have been explored to check how robust the SampEn measures against these small changes in parameters.

#### Permutation Entropy

Bandt and Pompe (2002) developed PEn to determine the occurrence of ordinal patterns in time series data. The PEn is a robust and straightforward measure that quantifies the regularity of a time series by comparing neighboring values to estimate the intrinsic structures in EEG data (Bandt and Pompe, 2002). Thus, for the time series *x(i)* of length *N*, the normalized PEn is given by:


(8) $$P\,E\,n=-\frac{\sum_{i=1}^{n}p_{i}\,l\,o\,g\,p_{i}}{l\,n\,\left(n\right)}$$

where *n* is the order pattern, and *p*_*i*_ is the probability of the *ith* permutation occurring. The smaller the value of PEn, the more regular the time series is.

The appropriate selection of embedding parameters, including dimension *m* and time delay *L*, is necessary for proper PEn estimation. For this purpose, Olofsen et al. (2008) suggested the values of *m* = 3, and *L* = 1–2. In this exploration, the PEn was computed using these recommended values to investigate whether the small changes in these parameters could affect the PEn estimation.

#### Spectral Entropy

Spectral entropy is another common EEG complexity measure that computes the randomness of the power spectrum of a signal (Li et al., 2008). Thus, unlike SampEn and PEn, SpEn measures the signal irregularity in the frequency domain. For this end, SpEn applies the Shannon entropy concept to the normalized power spectral density (PSD) of the signal such that,


(9) $$S\,p\,E\,n=-\sum_{i=1}^{N}p_{i}\,\log p_{i},$$

where *p*_*i*_ is the probability distribution of PSD at each frequency points and N is the total frequency points. SpEn was calculated using the frequency range 0.5–45 Hz. The frequency range was selected to focus on the range of interest with respect to traditional brain waves.

Spectral entropy is an efficient method to reflect the degree of skewness in the frequency distribution. A high value of SpEn indicates a flat, uniform spectrum with a broad spectral content, and a low value of SpEn describes a spectrum with all the power condensed into a single frequency point (Cui et al., 2015).

### Features Extraction Procedure

The features extraction stage in the proposed analysis was divided into two parts. The first phase focused on decomposing EEG signals into their intrinsic components by using NA-MEMD. The second part involved extracting the two fundamentally different classes of features, namely graph-theoretical attributes and entropies features.

#### Step 1: Noise-Assisted Multivariate Empirical Mode Decomposition Analysis

**FIGURE 3:**
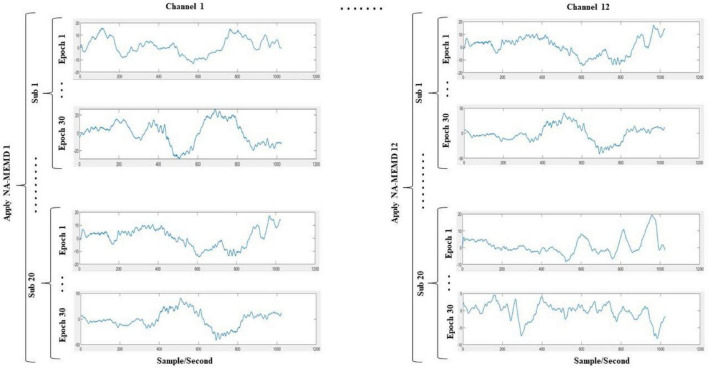
Proposed simultaneous decomposition method of the EEG signals. The data points of all infants (including all epochs) are stacked on top of each other. This process is done for each channel separately, ending up with 12 multivariate signals; each of them has the dimensionality of [*N*_*s*_×*N*_*e*_]×*N*_*t*_, where *N_s_* denotes the number of infants (which is 20), *N_e_* is the number of epochs of each infant (which is 30), and *N_t_* indicates the number of temporal samples (which is 1024). The NA-MEMD is then applied for each of the 12 multivariate signals separately.

**FIGURE 4:**
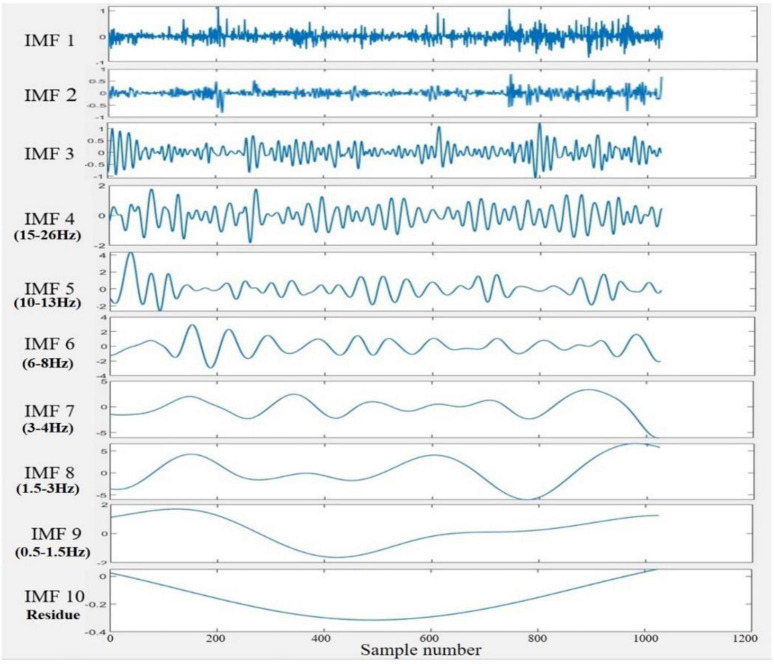
An example of a set of IMFs resulted from the NA-MEMD decomposition of the 2 s EEG signal. IMF1 to IMF3 considered noisy, and IMF10 represented the residue mode. IMF4 to IMF6 were localized in the beta, alpha, and theta bands, respectively, while IMF7 to IMF9 belonged to the delta band.

1. A multivariate signal was constructed by combining the data points from all infants for each channel separately. The idea of combining signals from different sources decomposing them using NA-MEMD to acquire aligned IMFs was previously conducted in the literature (Zahra et al., 2010). This yielded twelve different matrices (i.e., one matrix per channel); each of them has the dimensionality of *N*_*s*_×*N*_*t*_×*N*_*e*_,where *N_s_* denotes the number of subjects (which is 20), *N_t_* indicates the number of temporal samples (which is 1024), and *N_e_* is the number of epochs of each subject (which is 30).
2. Before decomposing the twelve multivariate signals by the NA-MEMD algorithm, each matrix was set up in a two-dimensional time series of the dimensions [*N*_*s*_×*N*_*e*_]×*N*_*t*_. Therefore, the alignment among all IMFs across infants and over epochs was ascertained. A similar approach has been used previously by Hu and Liang (2011). Figure 3 illustrates the decomposition process.
3. The resulting IMFs components after the decomposition process were slightly varied between channels. Since the EEG channel that yields the lowest number of IMFs upon decomposition gives ten modes, the first ten IMFs of each channel were considered for feature extraction. Figure 4 depicts the sample of extracted IMFs from a channel that gave ten IMFs.
4. The frequencies of each IMF were then acquired by fast Fourier transform (FFT), and it was found that IMF1 to IMF3 are noisy and contain different oscillatory components. Thus, these modes were excluded from further analysis. IMF10 was also ignored as it represented the residue mode of some EEG channels, which might give unreal information about the signal. The scales of the remaining IMFs were localized approximately around the following ranges: IMF4 (15–26 Hz), IMF5 (10–13 Hz), IMF6 (6–8 Hz), IMF7 (3–4 Hz), IMF8 (1.5–3 Hz), and IMF9 (0.5–1.5 Hz). Compared to five standard brain waves (Sanei and Chambers, 2007): IMF4 to IMF6 belong to beta, alpha, and theta bands, respectively; IMF7 to IMF9 all correspond to the delta band.
5. After selecting the IMFs, a dataset of the following dimensionality for each subject was achieved: *N*_*c*_×*N*_*i*_×*N*_*e*_×*N*_*t*_,where *N_c_* is the number of channels which is 12, *N_i_* is the selected number of IMFs which is 6 (IMF4–IMF9), *N_e_* is the number of epochs which is 30, and *N_t_* is the number of the samples which is 1024.

### Step 2: Feature Extraction

#### Weighted Phase-Lag Index

Before extracting the five graph-theoretical features, each IMF frequency scale alignment among channels was checked. Following that, the WPLI connectivity matrix was calculated between twelve channels for each IMF and each epoch. The generating WPLI matrices were averaged over the epochs, yielding one averaged connectivity matrix for each IMF. Then, each connectivity matrix was transformed into a complex connectivity network, and the graph-theoretical parameters were estimated to quantify its properties. The extracted graph parameters were then used to train and test the models.

#### Entropy Features

For each channel and each IMF, the proposed entropy measures were computed for each epoch. The calculated features were then averaged among the epochs to get one SampEn, one PEn, and one SpEn for each IMF signal. Thus, for each subject and each IMF signal, we ended up with 36 features (3 features × 12 channels). These features were then used to train and test the regression models.

### Statistical Analysis: Correlation Coefficient

Correlation analysis was used to statistically measure the strength of the relationship between two random variables. There are several types of correlation methods, such as Pearson, Spearman, and Kendall. Pearson’s correlation coefficient was adopted herein to evaluate whether there is a linear relationship between the proposed qEEG features and the cognitive scores. Although it is sensitive to outliers, Pearson’s coefficient is chosen as it is the most widely used technique to measure the linear relationship between two variables, easy to compute and simple to interpret.

Pearson correlation coefficient, denoted by r, was adopted herein for this purpose. Theoretically, the value of r falls in the interval between +1 and −1, with 0 indicates no linear relationship, +1 refers to a perfect positive correlation, i.e., when one variable increases, the other increases too, while −1 indicates a perfect negative correlation, i.e., when one variable increases, the other decreases.

The significant test was conducted through the hypothesis test to evaluate the significance of the correlation coefficient. *P*-value assesses the null hypothesis that stated that there is no relationship between qEEG features and cognitive scores. The null hypothesis is successfully rejected when the *p*-value is below the significant level, which usually equals 0.05, indicating that the correlation coefficient result is statistically significant.

*P*-value is generally calculated based on t-statistic using the following equation:


(10) $$t=\frac{r\times \sqrt{n-2}}{\sqrt{1-r^{2}}},$$

where r is the correlation coefficient, and n is the size of the dataset. Then the *p*-value is calculated from the t-distribution.

The correlation analysis was performed using MATLAB’s statistics toolbox.

Thus, for the graph-theoretical of WPLI, the correlation coefficient was utilized to determine the correlation strength between the five graph-theoretical features and the cognitive scores in each IMF. The *p*-value was also calculated to identify the high influence predictors (qEEG features).

For entropy analysis, the correlation coefficient was performed to determine the linear dependency between each entropy feature (SampEn, PEn, and SpEn) computed from each channel and the cognitive scores. The *p*-value was also computed to evaluate the statistically significant predictors. This process was repeated for each IMF separately.

### Regression Model

A regression model was used to predict the later cognitive scores of the infants. The model tries to fit the relationship between the two proposed sets of EEG features (graph metrics and entropies) and the cognitive scores with the least possible error. The tree ensemble regression models were adopted in this study in order to reduce bias and variance in the imbalanced distribution of our dataset–as the distribution of the cognitive scores ranged between 74 and 145, such that most of the scores clustered above 95.

The basic idea of tree ensemble regression is using several combined models to obtain improved predictive performance (Moniz et al., 2017). Boosted trees regression and bagged trees regression were the two ensembles’ models adopted in this study. Bagged tree regression randomly sampled the original dataset into different subsets with replacement. Several homogeneous models run independently on each subset in parallel, and the final predictive performance is obtained by combining the estimations of several models. In contrast, the boosted tree is a sequential ensemble method in which several homogenous models train adaptively. Each example in the dataset is assigned with weight. The examples that are incorrectly classified carry more weight than the examples that are correctly classified. Thus, the successor classifier focuses more on the example with the high weight that the predecessor classifier failed to classify correctly. A more detailed description of these models is available in Moniz et al. (2017) and MathWorks (2021).

Tree ensembles regression models were trained with both proposed sets of features separately. Regression learner apps within the statistics and machine learning toolbox in MATLAB was used to train the selected models.

Leave-one-subject-out cross-validation (LOSOCV) was used to assess the model performance in order to avoid the biased estimation of the prediction performance. This method works by splitting the dataset into two parts: one used for training and the other for testing. The training set contains *N*-1 individuals (where *N* = 20), and the remaining individual is turned into the testing set. Each individual is left out once in an iterative framework (*N* iterations), and then model statistics are evaluated by averaging the *N* independent regression outcomes.

The performance of regression models was evaluated by the traditional measures known as root mean square error (RMSE), mean absolute error (MAE), and *R*-squared. RMSE is the most frequently used metric. It refers to the square root of the average squared difference between the predicted score resulting from the regression model and the actual one. Lower RMSE indicates the better model’s performance. MAE is the absolute difference between the predicted value and the target one, and as in the case of RMSE, the lowest value refers to the best model’s performance. *R*-squared is another metric used to evaluate the performance of the regression model. It determines how well the model predicts the specific score by comparing the learned model with the constant baseline model. The constant baseline model is built by taking the mean of training data and drawing the line on the mean. The value of *R*-squared is usually less than or equal to 1 where the higher value refers to a better fit between predicted and actual values.

## Results

### Weighted Phase-Lag Index-Based Functional Brain Connectivity Results

**FIGURE 5:**
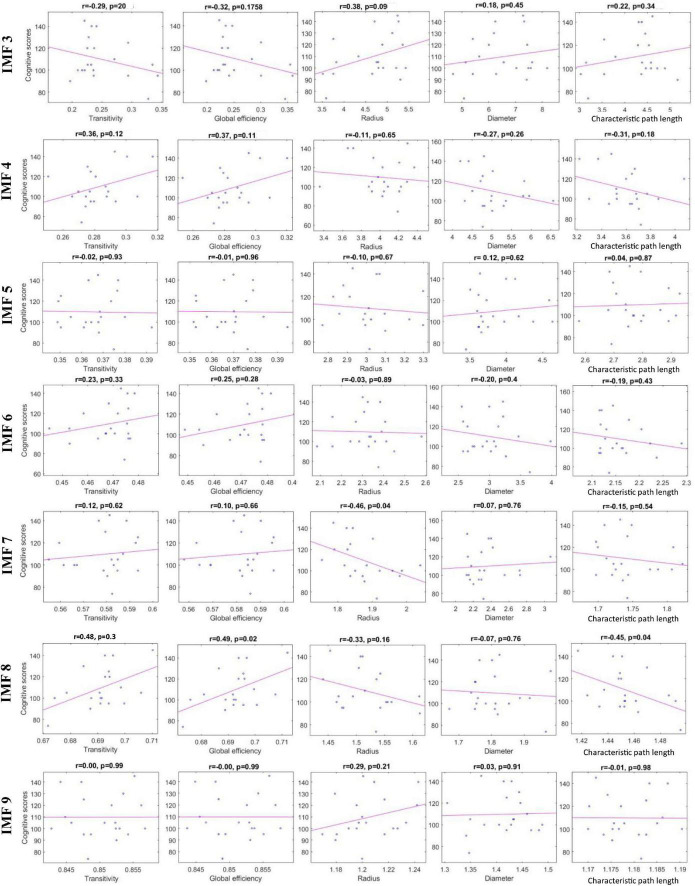
Scatter plots representing the correlation between the graph-theoretical features calculated from each IMF and cognitive scores.

Table 3 shows the *p*-value results of the correlation analyses between graph-theoretical features and cognitive scores in each IMF component. In Table 3, features with the smallest *p*-value are shown with boldface, indicating the statistically significant correlation with the cognitive scores. These features were radius calculated from IMF7 (3–4 Hz) and transitivity, global efficiency, and characteristic path length computed from IMF8 (1.5–3 Hz). Correlation plots in Figure 5 reveal that the radius and characteristic path length exhibit a significant negative correlation (*r* = −0.46, *p* = 0.04) and (*r* = −0.45, *p* = 0.04), respectively. Transitivity and global efficiency show a high positive correlation (*r* = 0.48, *p* = 0.03) and (*r* = 0.49, *p* = 0.02), respectively.

**TABLE 3:** *P*-values of the correlation analysis of the graph-theoretical features.

	IMF4 (15–26 Hz)	IMF5 (10–13 Hz)	IMF6 (6–8 Hz)	IMF7 (3–4 Hz)	IMF8 (1.5–3 Hz)	IMF9 (0.5–1.5 Hz)
**Transitivity**	0.12	0.93	0.33	0.62	**0.03**	0.99
**Global efficiency**	0.11	0.96	0.28	0.66	**0.02**	0.99
**Radius**	0.65	0.67	0.89	**0.04**	0.16	0.21
**Diameter**	0.26	0.63	0.4	0.76	0.76	0.9
**Characteristic path length**	0.18	0.87	0.43	0.54	**0.04**	0.9

*Significant features are shown in boldface.*

Considering that these features have highly significant correlation coefficients, they could greatly influence predicting the cognitive outcome. Thus, these four features were selected to be used in training and testing the regression models.

**FIGURE 6:**
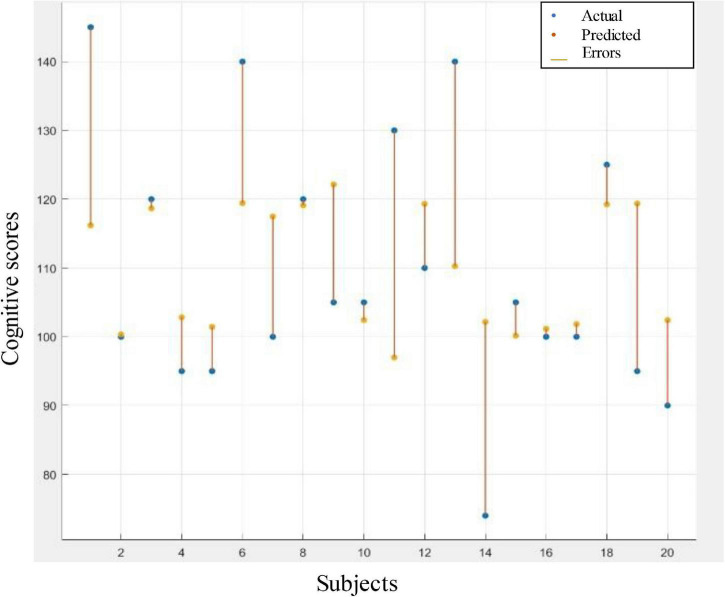
Response plot of predicted cognitive scores versus the actual one. Regression based prediction using radius graph feature to predict the cognitive scores.

LOOCV was used to evaluate the models’ performance to prevent potential bias from occurring due to overfitting. Table 4 depicts the performance of tree ensemble regression models of the four selected features and their combinations. It is apparent from Table 4 that the best performance–in terms of lowest RMSE, MSA, MAE, and highest *R*-squared–was achieved using radius network property from IMF7 (3–4 Hz). The visualization corresponding to this result represented by the difference between predicted scores and the actual scores is shown in Figure 6. The error rate between the predicted values and actual ones of majorities of the individual was acceptable as depicted in Figure 6. Other features such as transitivity, global efficiency and characteristic path length calculated from IMF8 (1.5–3 Hz) also give comparable results. This result implies that the network attributes–mainly radius–could provide valuable information regarding cognitive outcomes.

**TABLE 4:** Performance of the tree ensembles regression models using the significant graph-theoretical features.

Scale	Feature	RMSE	MAE	*R*-squared	Regression algorithm
IMF7 (3–4 Hz)	Radius	**16.775**	**12.7**	**0.24**	**Bagged trees**
		18.945	14.2	0.03	Boosted trees
IMF8 (1.5–3 Hz)	Transitivity	17.317	13.64	0.19	Bagged trees
		17.802	13.86	0.15	Boosted trees
	Global efficiency	17.26	13.64	0.2	Bagged trees
		17.71	13.82	0.15	Boosted trees
	Characteristic path length	16.98	13.28	0.22	Bagged trees
		17.78	13.85	0.15	Boosted trees
	Combination of transitivity, global efficiency, and characteristic path length	17.11	13.23	0.21	Bagged trees
		17.842	13.897	0.14	Boosted trees

*The best model performance is shown in boldface.*

### Entropy Analysis Results

Correlation analyses were carried out between the entropy measures, computed from each IMF and each channel, and the cognitive scores of all participants. Different embedding dimensions *m*, tolerances *r*, and time delay *L* were explored for SampEn and PEn estimations as suggested by Bruhn et al. (2000); Tudor et al. (2005); Olofsen et al. (2008). The correlation results indicate the robustness of SampEn and PEn with the small changes of the embedding parameters. Table 5 presents the *p*-values of the correlation analyses using *m* = 3, *r* = 1, and *L* = 1 for SampEn computation, and *m* = 3 and *L* = 1 for PEn calculation. The detailed correlation values of other embedding parameters are presented in the Supplementary Table.

**TABLE 5:** *P*-values of the correlation analysis of the complexity features.

	Channel index	IMF4 (15–26 Hz)	IMF5 (10–13 Hz)	IMF6 (6–8 Hz)	IMF7 (3–4 Hz)	IMF8 (1.5–3 Hz)	IMF9 (0.5–1.5 Hz)
**SampEn**	C3	0.79	0.97	0.95	0.56	0.51	0.19
	F3	0.42	0.86	0.77	0.28	0.40	0.66
	F7	0.86	0.41	0.95	0.99	0.83	0.50
	Fz	0.38	0.61	0.07	0.13	0.71	0.27
	O1	0.61	0.45	0.41	0.93	0.32	0.43
	O2	0.27	0.52	0.97	0.50	0.07	0.81
	P3	0.33	0.75	0.33	0.68	0.42	0.71
	P4	0.33	0.92	0.68	0.46	0.86	0.18
	T3	0.59	0.42	0.82	0.18	0.98	0.84
	T4	0.73	0.77	0.66	0.51	0.79	0.48
	T5	0.28	0.65	0.60	0.74	0.68	0.27
	T6	0.50	0.71	0.48	0.32	0.95	0.07
**PEn**	**C3**	0.68	0.80	0.30	0.79	0.27	**0.01**
	F3	0.32	0.52	0.69	0.24	0.54	0.27
	F7	0.27	0.48	0.42	0.56	0.63	0.12
	Fz	0.39	0.47	0.37	0.32	0.81	0.89
	O1	0.49	0.39	0.09	0.98	0.85	0.95
	O2	0.18	0.98	0.66	0.80	0.62	0.27
	P3	0.31	0.84	0.86	0.26	0.76	0.73
	P4	0.12	0.42	0.93	0.47	0.65	0.37
	T3	0.49	0.33	0.51	0.15	0.40	0.28
	T4	0.88	0.33	0.52	0.24	0.99	0.28
	T5	0.13	0.92	0.44	0.56	0.61	0.24
	T6	0.59	0.89	0.48	0.73	0.43	0.40
**SpEn**	C3	0.49	0.49	0.99	0.42	0.44	0.74
	F3	0.55	0.55	0.90	0.08	0.12	0.93
	F7	0.83	0.83	0.10	0.99	0.21	0.55
	Fz	0.56	0.56	0.30	0.42	0.85	0.69
	O1	0.56	0.56	0.39	0.90	0.56	0.57
	O2	0.96	0.96	0.94	0.16	0.14	0.85
	P3	0.40	0.40	0.69	0.53	0.89	0.12
	P4	0.72	0.72	0.57	0.69	0.55	0.47
	**T3**	0.60	0.60	0.74	0.26	0.62	**0.05**
	T4	0.65	0.65	0.78	0.88	0.24	0.73
	**T5**	0.69	0.69	0.75	0.14	0.26	**0.03**
	T6	0.78	0.78	0.68	0.96	0.73	0.11

*Significant features are shown in boldface.*

The results presented in Table 5 show that the significant *p*-values generally realize in the IMF9 (0.5–1.5 Hz) component of the left cerebral hemisphere. Particularly, the PEn calculated from channel C3 and SpEn computed from channels T3 and T5 exhibit significant correlations with the vector of the cognitive scores.

**FIGURE 7:**
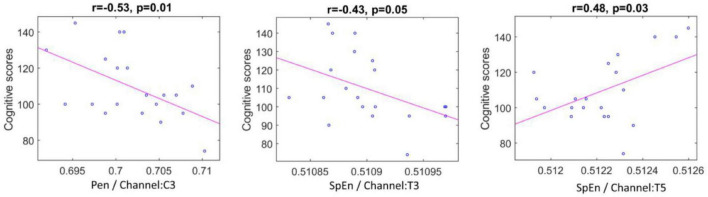
Scatter plots representing correlation between the significant entropy features and cognitive scores.

According to the correlation plots in Figure 7, the PEn feature of channel C3 and SpEn feature of channel T3 are shown to exhibit a significant negative correlation of (*r* = −0.53, *p* = 0.01) and (*r* = −0.43, *p* = 0.05), respectively. In addition, a high positive correlation is demonstrated by the SpEn of channel T5 (*r* = 0.48, *p* = 0.03). Thus, these features were selected to be used in the regression stage.

**FIGURE 8:**
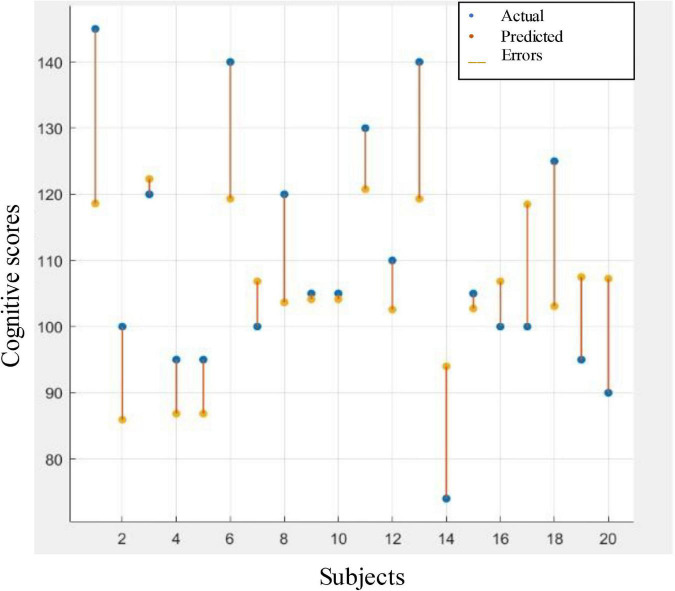
Response plot of predicted cognitive scores versus the actual one. Regression based prediction used the combination of the selected entropies features to predict the cognitive scores.

Table 6 illustrates the performance of LOSOCV when the models were trained and tested on the combination of the selected entropies. It can be noticed from the table that the boosted tree regression model achieved the best performance. Figure 8 gives the visualization corresponding to this result. The figure reveals acceptable error rates, for most individuals, between the predicted values and the actual ones. This result indicates a reasonable prediction value for the cognitive scores using PEn and SpEn extracted from the IMF9 (0.5–1.5 Hz) component corresponding to the delta band. Further, we can infer that a good predictive value of the cognitive outcome is observed from the deterioration of EEG complexity produced by the left hemisphere.

**TABLE 6:** Performance of the tree ensemble regression models using the significant entropies features computed from IMF9 (0.5–1.5 Hz).

Features	RMSE	MAE	*R*-squared	Regression algorithm
PEn (C3)	17.069	14.025	0.21	Bagged tree
	16.856	14.151	0.23	Boosted tree
SpEn (T3)	17.993	15.116	0.13	Bagged tree
	17.876	13.823	0.14	Boosted tree
SpEn (T5)	18.789	14.26	0.05	Bagged tree
	19.957	15.058	0.07	Boosted tree
Combination of PEn (C3), SpEn (T3) and (T5)	16.283	12.655	0.29	Bagged tree
	**14.271**	**12.067**	**0.45**	**Boosted tree**

*The best model performance is shown in boldface.*

## Discussion

The objective of the present investigation is to explore the effectiveness of employing qEEG analysis in the early prediction of cognitive outcome, assessed at 2 years of age following neonatal HIE. The early phase of a child’s life is considered a critical stage for cognition, motor, language and social-emotional development owing to brain development and maturation of cortical architecture that are most rapidly established in this period (Ouyang et al., 2020). Early identification of the infants who have the cognitive impairment could help to provide a tailored intervention seeking to improve the outcome by utilization of this property of the brain which is called brain plasticity.

Two sets of features have been adopted for early identification of high-risk infants to develop cognitive impairment at 2 years of age, which were graph attributes of WPLI and complexity features extracted from EEG signals of twenty infants with neonatal HIE during their first week after birth.

The most significant challenge encountered in this study was that the distribution of the dataset was biased, with most cognitive scores clustered above 95, within 1 SD from the population mean. Most of the efforts in the machine learning community have been devoted to eliminating current challenges by designing an algorithm that can deal with bias and variance in the dataset. Tree ensembles regression, employed herein, is one of the efficient algorithms developed to handle this problem. It is designed to train multiple models and then combine their results to improve the performance of the final model. Furthermore, the subjective nature of the employed pre-processing techniques may also be considered as some artifacts still need to be removed using visual inspection. Hence, a fully automated process is of great interest to avoid subjective biases.

To the best of our knowledge, this research constitutes the first analysis on the impact of both proposed qEEG features calculated in the NA-MEMD domain for predicting cognitive outcome. Graph-theoretical metrics of WPLI were the first class of features adopted in this study. Statistical analysis shows a a significant correlation between the graph-theoretical features and the cognitive scores in the delta band connectivity corresponding to IMF7 (3–4 Hz) and IMF8 (1.5–3 Hz) components using radius, characteristic path length, transitivity, and global efficiency attributes. A strong negative relationship between radius and cognitive profiles is observed, indicating that the trend of the higher radius is correlated with poorer cognitive outcomes. This result suggests that the weak connections in brain networks (represented by increase in the radius) is negatively related to cognitive performance, i.e., increase in radius of the brain network is associated with poorer cognitive development.

On the other hand, a negative correlation is revealed in characteristic path length (a measure of network efficiency) with cognitive scores, displaying that an increase in characteristic path length is inversely associated with cognitive scores. This result indicates that a less efficient brain network in terms of global information transfer (measured by increase in characteristic path length) is negatively correlated with the cognitive level, i.e., increase in characteristic path length conducive to a reduction in the cognitive performance.

Moreover, the correlation analysis demonstrates a positive relationship between transitivity and global efficiency, and cognitive outcome, i.e., high transitivity and global efficiency, yielding higher cognitive scores. These results indicate that network efficiency in terms of information transfer between different brain regions is positively associated with cognitive outcome, i.e., increase in transitivity and global efficiency leads to better cognitive outcomes.

To sum up, the global graph-theoretical metrics (except diameter) appear promising to act as biomarkers for early prediction of cognitive outcome. This finding is consistent with existing studies other conditions. For example, the study by Peters et al. (2013) showed study showed increased characteristic path length and decreased global efficiency in brain networks of children with Autistic Spectrum Disorder (ASD).

The second class of features employed in this study were entropy measures, which are traditionally used to estimate the degree of EEG complexity. The existing entropy measures quantify the regularity of a time series represented on a single scale (Takahashi et al., 2010), Multiscale entropy (Costa et al., 2002) which measures the complexity considering different scales inherent in the signal. Though powerful, the multiscale entropy method is not well suited for studying non-linear and non-stationary signals due to its linear extraction of scale (Peng et al., 2009). Subband wavelet entropy (SWE) (Al-Nashash et al., 2003) was also proposed to measure Shannon entropy from multiscale components. However, SWE is based on the wavelet method, which relies on predefined frequency ranges for the decomposition process. In the present work, we addressed these issues by computing the proposed entropies over different signal scales/IMFs extracted by NA-MEMD, which decomposes the signals adaptively and considering the non-linear and non-stationary nature of EEGs (Hu and Liang, 2011; Looney et al., 2015). The correlation analysis revealed a significant association in the delta band component, corresponding to IMF9, between entropies and the cognitive scores. Particularly, a significant positive correlation is observed between the SpEn extracted from the posterior brain area and the cognitive scores. This correlation indicates that the lower SpEn in this region was associated with a higher potential of cognitive impairments and vice versa. This result follows the general assumption of the physiological complexity reduction being related to various pathological processes (Catarino et al., 2011; Chu et al., 2017). A positive positive correlation between complexity measures and several cognitive functions at multiple brain regions was reported in different studies in older adults (Hu et al., 2016).

The analysis also demonstrates a negative correlation between the Pen, SpEn computed from the central brain region and the cognitive outcome. This result shows that the randomness behavior of the brain is negatively related to the level of cognitive function, i.e., an increase in the complexity of EEG signals leads to a reduction in the cognitive performance. This finding contradicts the general assumption of decreased complexity in a damaged brain. Li et al. (2010) reported a similar increase in the ApEn neurologically abnormal neonates with HIE or epilepsy compared to normal term neonates. Sajedi et al. (2013) also showed higher complexity in individuals with CP and reported that the CP neuronal networks functions are more random than in a normal brain. They indicated that this increase in complexity might result from reduced coordination among the neuronal regions in the disordered brain. This interpretation could be traced back to the non-linear dynamical theory, where it is known that non-linear coupled oscillations exhibit enhancement of complexity by the emergence of a chaotic state while reducing the coupled strength for the state of complete synchronization (Pikovsky et al., 2001). Thus, during decreasing coupled strength, the synchronization reduces, and the complexity enhances. Following this hypothesis, the decrease of entropy measures at the central region could be explained by the changes in the interactions of that brain area and other areas.

Nevertheless, the clinical significance of such inconsistent results remains unclear. Still, the findings indicate that the complexity measures of EEG could, at least, be useful in identifying abnormal brain function.

The correlational analyses also suggest that cognitive development of infants at the age of 2 years is associated with EEG complexity of the left hemisphere. Although several studies claimed that the right hemisphere is dominant in infants (Chiron, 1997; Adibpour et al., 2018), Chiron (1997) reported that the right hemisphere sustains the visuospatial abilities while the left hemisphere dominance for language function. Moreover, socio-cognitive function has also been linked by different studies to the neurophysiological processes of the left-brain. For example, there is ample evidence that relatively higher left than right frontal activity is related to social behavior (Harmon-Jones et al., 2010). Paulus et al. (2013) showed that prosocial understanding is associated with relatively stronger left frontal cortical activation in 2 years old infants. The study by Kühn-Popp et al. (2016) supported the socio-cognitive function of left-hemispheric brain maturation processes, which proved to be the prominent independent predictor of social communication abilities at 48 months.

Both proposed methods (WPLI and complexity analysis) reveal correlations between neonatal EEG characteristics and cognitive outcome in the delta band. This finding is consistent with Suppiej et al. (2017), where the authors concluded that the high value of the delta power spectral correlated with poor outcomes in preterm infants during the first year of age. Increased delta activity in EEG of children suffering from learning disorders was also confirmed by Martínez-Briones et al. (2020) and further support by Barttfeld et al. (2011) who reported the difference in delta band coherence between children having ASD and a control group. However, some studies have found the alterations in theta band waves in infants is a predictive biomarker for later cognitive impairment during the presenting of a specific stimulus. These studies suggested change in theta waves in infants when the subject tried to respond to an external stimulus. Jones et al. (2020) reported that EEG theta change in infancy during the presentation of dynamic movies of people and objects is a predictive biomarker for later cognitive deficits, particularly in high-risk populations. Braithwaite et al. (2020) also found increases in the theta wave recorded at 6-month-old infants at low-risk while presenting the non-social video. These changes in theta significantly predicted non-verbal cognitive ability measured at age 9-months. Begus et al. (2015) found that increases in frontal theta oscillations during object exploration correlated with subsequent recognition of that object in infants aged 11 months. The heterogeneity of the brain waves that play essential roles in the cognitive development of the infants can be attributed to the differences in the experimental conditions. While most studies that found correlations between brain characteristics and cognitive functions in the delta band used resting-state EEG, the research that reported associations between EEG features and their corresponding cognitive profile in the theta wave have employed task-related EEG analysis.

Two tree ensembles regression models were explored to handle the bias distribution of our dataset. The significant graph-theoretical features (transitivity, global efficiency, radius and characteristic path length) and complexity features (SpEn and PEn) calculated from IMF components corresponding to the delta band were used to train and test the regression models. The best performance has been observed using boosted tree regression with a combination of Pen and SpEn features from left-brain regions (root mean square error score (RMSE) = 14.27, MAE = 12.07, and *R*-squared = 0.45). A comparable result was also observed using bagged tree regression with radius as a feature (RMSE = 16.78, MAE = 12.07, and *R*-squared = 0.24).

A key strength of this research was recognized when compared with the state-of-the-art of qEEG studies, shown in Table 7. To the best of our knowledge, our study is the first prospective study to date performed in neonates (in the first week of birth) investigating early non-linear qEEG characteristics (WPLI and complexity measures) and their prognostic value for cognitive outcome at 24 months of age. Furthermore, all studies existing in literature have used the linear qEEG such as coherence (Kühn-Popp et al., 2016), EEG continuity (West et al., 2005), and spectral power (Suppiej et al., 2017; Cainelli et al., 2021), which may not be optimal to capture the complex characteristics of the EEG spectra. Non-linear methods adopted in this study provided deep insight into the underlying brain functions and dynamics.

**TABLE 7:** Comparison of the qEEG state-of-the-art methods employed for predicting cognitive outcomes.

Author	Dataset	Features	Evaluation methods	Outcome assessment	Findings
Lloyd et al., 2021	57 preterm infants	EEG grading	Spearman’s correlation coefficient	BSITD-III	Moderate to large negative correlation between EEG grade and Bayley-III subscale
Suppiej et al., 2017	21 preterm infants	Power spectral analysis	Spearman’s correlation coefficient	Griffiths Scale of Mental Development	Negative correlation between the delta spectral power and Griffiths scores developmental quotients (*r* = −0.68, *p* = 0.015). Positive correlation between alpha and beta power spectral and Griffiths developmental quotients (*r* = 0.61, *p* = 0.032).
Cainelli et al., 2021	26 preterm infants	Power spectral analysis	Bayesian correlation	Wechsler Pre-school and Primary Scale of Intelligence III (WPPSI-III) test	Significant association between spectral frequency bands and visual and auditory attention tests.
West et al., 2005	44 preterm infants	EEG continuity	Linear regression	BSITD-II	Significant correlation between mental developmental indices and continuity feature of EEG at different amplitude setting: 10 and 25 μV thresholds (*R*-squared = 0.19, *P* = 0.0032 and *R*-squared = 0.10, *p* = 0.04, respectively).
Kühn-Popp et al., 2016	32 infants	EEG coherence measures	Linear regression	Coding-scheme for mental state terms	Significant correlation between left hemisphere coherence and epistemic language at 48 months (*r* = 0.59, *p* = 0.003). Regression analyses showed, left-coherence scores are the most important predictor of epistemic state talk at 48 months
Current study	20 infants born with HIE	● Graph-theoretical features derived from WPLI ● Entropy features	● Pearson linear correlation coefficient ● Set of regression models	BSITD-III	**Connectivity:** significant correlation between transitivity, global efficiency, radius, and characteristic path length and cognitive outcomes. Reasonable regression performance with radius feature: RMSE (16.775), MAE (12.702), and *R*-square (0.24). **Complexity:** significant correlation between PEn and SpEn measured from left regions of brain and cognitive profile. Good regression performance with combination of PEn from C3 and SpEn T3 and T5: RMSE (14.27), MAE (12.07), and *R*-square (0.45).

Several studies investigated the frequency activity underlying EEG by spectrally analyzing the signal using methods that rely on the predefined frequency of traditional brainwaves, as in Kühn-Popp et al. (2016), Suppiej et al. (2017), and Cainelli et al. (2021). Prior selection of frequency ranges may result in potentially informative brain waves being missed, specifically in the case of infants, due to the well-known variability between them and older individuals in the neural oscillations of interest. Moreover, the predefined basis may not be able to fit all the non-linear and non-stationary phenomena (Huang et al., 1998). This constraint has been settled in our proposed approach using the NA-MEMD method, which decomposes the signals adaptively. Thus, with this method, we ascertained that all meaningful brain dynamics are included in the analysis, and no misleading energy-frequency distribution will result from analyzing the non-stationary and non-linear signals. Nevertheless, the advantages of the EMD-based methods have a price of being empirically, not theoretically, defined. In addition, the NA-MEMD method has a very high computational complexity having a subspace of multivariate independent white noise equal to the original multivariate signal.

In summary, this study provided a regression-based machine learning framework to objectively predict cognitive outcome at toddler age, and a good performance was achieved. We found that the global network attributes and entropy features could serve as early markers for cognitive development. Further, the experimental results found a association between neonate’s left-brain region and cognitive deficit emerging at 2 years. Due to the limited dataset size, this study can only be considered a proof of concept work. It is provided the initial proof of that employing the qEEG features within regression-based machine learning frameworks could capture the individual variability inherited in infants’ developing brains. This study lays the groundwork for future investigations into using these features as the potential biomarkers for predicting cognitive development in a number of populations who are at risk for long term cognitive impairment or intellectual disability. This could assist in establishing tailored intervention programmes at an early stage to improve outcomes. Nevertheless, a further refinement of the proposed analysis with larger sample sizes is required to validate the findings.

## Conclusion

In this investigation, the analysis of qEEG successfully predicted the cognitive outcome at 2 years of age in infants with neonatal HIE. Complexity analysis (SampEn, PEn, and SpEn) and functional brain connectivity (WPLI) measures were evaluated by correlation analysis and regression-based machine learning framework. Pearson linear correlation analysis showed a strong correlation between graph-theoretical features of WPLI, PEn, SpEn, and cognitive scores. The tree ensembles regression models have achieved comparable performance in both methods, with relative superiority of complexity measures using combination between PEn and SpEn; RMSE (14.27), MAE (12.07), and *R*-square (0.45). Therefore, the findings of this study have provided insight into the possibility of using graph-theoretical features and entropy measures derived from the delta band as biomarkers for early prediction of cognitive development.

## Data Availability Statement

The raw data supporting the conclusions of this article will be made available by the authors, without undue reservation.

## Ethics Statement

The studies involving human participants were reviewed and approved by HRA and Health and Care Research Wales, HCRW (Reference ID 20/HRA/0260; IRAS project ID 278072; University Hospital Southampton R&D protocol number RHM CHI1047). Written informed consent to participate in this study was provided by the participants’ legal guardian/next of kin.

## Author Contributions

NA: methodology, software, formal analysis, data curation, writing–original draft, and visualization. DB: methodology, software, formal analysis, data curation, and writing–original draft. BV: providing the neurological evaluation information. DK: providing the EEG dataset. KM: conceptualization, validation, investigation, resources, and supervision. All authors contributed to the article and approved the submitted version.

## Conflict of Interest

The authors declare that the research was conducted in the absence of any commercial or financial relationships that could be construed as a potential conflict of interest.

## Publisher’s Note

All claims expressed in this article are solely those of the authors and do not necessarily represent those of their affiliated organizations, or those of the publisher, the editors and the reviewers. Any product that may be evaluated in this article, or claim that may be made by its manufacturer, is not guaranteed or endorsed by the publisher.
