# Supplementary material for: Cognitive Outcome Prediction in Infants With Neonatal Hypoxic-Ischemic Encephalopathy Based on Functional Connectivity and Complexity of the Electroencephalography Signal
Source: Front Hum Neurosci. 2022 Jan 27;15:795006. doi: 10.3389/fnhum.2021.795006 (PMC8830486; doi:10.3389/fnhum.2021.795006)
Supplement: Supplementary file 1 [file Table_1.DOCX]

Table I P-values of the correlation analysis of the SampEn features using different embedding parameters.

| **Embedding parameters** | **Channel** | **IMF4** | **IMF5** | **IMF6** | **IMF7** | **IMF8** | **IMF9** |
| --- | --- | --- | --- | --- | --- | --- | --- |
| **m=3, r=0.1** | C3 | 0.79 | 0.97 | 0.95 | 0.56 | 0.51 | 0.19 |
|  | F3 | 0.42 | 0.86 | 0.77 | 0.28 | 0.40 | 0.66 |
|  | F7 | 0.86 | 0.41 | 0.95 | 0.99 | 0.83 | 0.50 |
|  | Fz | 0.38 | 0.61 | 0.07 | 0.13 | 0.71 | 0.27 |
|  | O1 | 0.61 | 0.45 | 0.41 | 0.93 | 0.32 | 0.43 |
|  | O2 | 0.27 | 0.52 | 0.97 | 0.50 | 0.07 | 0.81 |
|  | P3 | 0.33 | 0.75 | 0.33 | 0.68 | 0.42 | 0.71 |
|  | P4 | 0.33 | 0.92 | 0.68 | 0.46 | 0.86 | 0.18 |
|  | T3 | 0.59 | 0.42 | 0.82 | 0.18 | 0.98 | 0.84 |
|  | T4 | 0.73 | 0.77 | 0.66 | 0.51 | 0.79 | 0.48 |
|  | T5 | 0.28 | 0.65 | 0.60 | 0.74 | 0.68 | 0.27 |
|  | T6 | 0.50 | 0.71 | 0.48 | 0.32 | 0.95 | 0.07 |
| **m=3, r=0.15** | C3 | 0.97 | 0.96 | 0.62 | 0.59 | 0.54 | 0.18 |
|  | F3 | 0.56 | 0.94 | 0.47 | 0.41 | 0.37 | 0.71 |
|  | F7 | 0.96 | 0.59 | 0.72 | 0.94 | 0.74 | 0.57 |
|  | Fz | 0.46 | 0.36 | 0.15 | 0.33 | 0.77 | 0.25 |
|  | O1 | 0.55 | 0.53 | 0.47 | 0.94 | 0.42 | 0.42 |
|  | O2 | 0.37 | 0.62 | 0.56 | 0.36 | 0.08 | 0.81 |
|  | P3 | 0.51 | 0.85 | 0.38 | 0.76 | 0.42 | 0.77 |
|  | P4 | 0.40 | 0.75 | 0.79 | 0.70 | 0.88 | 0.18 |
|  | T3 | 0.56 | 0.67 | 0.54 | 0.19 | 0.95 | 0.61 |
|  | T4 | 0.63 | 0.98 | 0.99 | 0.56 | 0.59 | 0.46 |
|  | T5 | 0.33 | 0.67 | 0.64 | 0.66 | 0.61 | 0.31 |
|  | T6 | 0.61 | 0.86 | 0.26 | 0.33 | 0.80 | 0.07 |
|  | C3 | 0.87 | 0.94 | 0.62 | 0.59 | 0.61 | 0.17 |
|  | F3 | 0.82 | 0.90 | 0.46 | 0.68 | 0.37 | 0.76 |
|  | F7 | 0.87 | 0.63 | 0.44 | 0.95 | 0.72 | 0.60 |
|  | Fz | 0.49 | 0.41 | 0.19 | 0.52 | 0.82 | 0.22 |
|  | O1 | 0.68 | 0.85 | 0.56 | 1.00 | 0.47 | 0.40 |
| **m=3, r=0.2** | O2 | 0.50 | 0.48 | 0.48 | 0.36 | 0.09 | 0.82 |
|  | P3 | 0.71 | 0.90 | 0.32 | 0.63 | 0.45 | 0.87 |
|  | P4 | 0.49 | 0.81 | 0.87 | 0.90 | 0.87 | 0.25 |
|  | T3 | 0.43 | 0.66 | 0.48 | 0.21 | 0.99 | 0.42 |
|  | T4 | 0.59 | 0.83 | 0.94 | 0.67 | 0.52 | 0.53 |
|  | T5 | 0.40 | 0.49 | 0.58 | 0.55 | 0.57 | 0.30 |
|  | T6 | 0.73 | 0.78 | 0.27 | 0.43 | 0.73 | 0.08 |
|  | C3 | 0.79 | 0.92 | 0.62 | 0.58 | 0.63 | 0.17 |
|  | F3 | 0.93 | 0.84 | 0.48 | 0.79 | 0.38 | 0.76 |
|  | F7 | 0.70 | 0.87 | 0.33 | 0.99 | 0.70 | 0.65 |
|  | Fz | 0.56 | 0.36 | 0.21 | 0.60 | 0.86 | 0.21 |
|  | O1 | 0.81 | 0.93 | 0.55 | 0.95 | 0.45 | 0.36 |
| **m=3, r=0.25** | O2 | 0.62 | 0.47 | 0.51 | 0.37 | 0.09 | 0.88 |
|  | P3 | 0.76 | 0.81 | 0.26 | 0.59 | 0.46 | 0.96 |
|  | P4 | 0.68 | 0.72 | 0.90 | 0.96 | 0.94 | 0.26 |
|  | T3 | 0.35 | 0.59 | 0.47 | 0.20 | 0.99 | 0.39 |
|  | T4 | 0.56 | 0.82 | 0.98 | 0.79 | 0.46 | 0.56 |
|  | T5 | 0.52 | 0.43 | 0.50 | 0.53 | 0.58 | 0.30 |
|  | T6 | 0.85 | 0.61 | 0.29 | 0.46 | 0.71 | 0.09 |
|  | C3 | 0.72 | 0.77 | 0.71 | 0.53 | 0.46 | 0.21 |
|  | F3 | 0.92 | 0.94 | 0.51 | 0.52 | 0.36 | 0.66 |
|  | F7 | 0.78 | 0.56 | 0.55 | 0.89 | 0.81 | 0.52 |
|  | Fz | 0.61 | 0.41 | 0.18 | 0.41 | 0.69 | 0.27 |
|  | O1 | 0.51 | 0.77 | 0.52 | 0.99 | 0.37 | 0.43 |
| **m=2, r=0.1** | O2 | 0.51 | 0.58 | 0.51 | 0.34 | 0.08 | 0.80 |
|  | P3 | 0.99 | 0.92 | 0.34 | 0.74 | 0.42 | 0.71 |
|  | P4 | 0.57 | 0.71 | 0.85 | 0.74 | 0.94 | 0.19 |
|  | T3 | 0.41 | 0.62 | 0.54 | 0.19 | 0.98 | 0.83 |
|  | T4 | 0.57 | 0.90 | 0.96 | 0.54 | 0.65 | 0.49 |
|  | T5 | 0.66 | 0.54 | 0.71 | 0.63 | 0.65 | 0.28 |
|  | T6 | 0.61 | 0.80 | 0.27 | 0.40 | 0.77 | 0.07 |
|  | C3 | 0.57 | 0.89 | 0.68 | 0.51 | 0.55 | 0.19 |
|  | F3 | 0.62 | 0.88 | 0.51 | 0.72 | 0.38 | 0.71 |
|  | F7 | 0.63 | 0.89 | 0.34 | 0.94 | 0.75 | 0.58 |
|  | Fz | 0.70 | 0.37 | 0.20 | 0.62 | 0.78 | 0.25 |
| **m=2, r=0.15** | O1 | 0.74 | 0.99 | 0.50 | 0.97 | 0.44 | 0.41 |
|  | O2 | 0.63 | 0.49 | 0.52 | 0.32 | 0.08 | 0.81 |
|  | P3 | 0.91 | 0.78 | 0.27 | 0.64 | 0.43 | 0.77 |
|  | P4 | 0.71 | 0.69 | 0.92 | 0.93 | 0.89 | 0.19 |
|  | T3 | 0.37 | 0.53 | 0.50 | 0.22 | 0.96 | 0.61 |
|  | T4 | 0.56 | 0.79 | 0.96 | 0.66 | 0.61 | 0.47 |
|  | T5 | 0.73 | 0.51 | 0.54 | 0.49 | 0.62 | 0.32 |
|  | T6 | 0.87 | 0.63 | 0.30 | 0.46 | 0.73 | 0.08 |
|  | C3 | 0.60 | 0.70 | 0.65 | 0.54 | 0.62 | 0.18 |
|  | F3 | 0.55 | 0.88 | 0.53 | 0.78 | 0.38 | 0.76 |
| **m=2, r=0.2** | F7 | 0.60 | 0.90 | 0.27 | 0.98 | 0.73 | 0.61 |
|  | Fz | 0.63 | 0.41 | 0.19 | 0.64 | 0.83 | 0.21 |
|  | O1 | 0.85 | 0.95 | 0.49 | 0.98 | 0.48 | 0.39 |
|  | O2 | 0.71 | 0.48 | 0.56 | 0.34 | 0.09 | 0.82 |
|  | P3 | 0.86 | 0.67 | 0.25 | 0.61 | 0.46 | 0.87 |
|  | P4 | 0.73 | 0.58 | 0.92 | 0.97 | 0.88 | 0.25 |
|  | T3 | 0.38 | 0.48 | 0.46 | 0.22 | 0.99 | 0.41 |
|  | T4 | 0.61 | 0.87 | 0.91 | 0.77 | 0.54 | 0.54 |
|  | T5 | 0.72 | 0.50 | 0.48 | 0.48 | 0.58 | 0.31 |
|  | T6 | 0.97 | 0.61 | 0.34 | 0.46 | 0.72 | 0.08 |
|  | C3 | 0.62 | 0.61 | 0.64 | 0.59 | 0.64 | 0.17 |
|  | F3 | 0.52 | 0.89 | 0.56 | 0.80 | 0.38 | 0.75 |
|  | F7 | 0.62 | 0.82 | 0.26 | 0.99 | 0.70 | 0.66 |
|  | Fz | 0.67 | 0.50 | 0.19 | 0.63 | 0.86 | 0.21 |
|  | O1 | 0.89 | 0.92 | 0.47 | 0.97 | 0.45 | 0.35 |
| **m=2, r=0.25** | O2 | 0.69 | 0.50 | 0.59 | 0.37 | 0.09 | 0.88 |
|  | P3 | 0.87 | 0.61 | 0.24 | 0.59 | 0.46 | 0.96 |
|  | P4 | 0.75 | 0.50 | 0.89 | 0.98 | 0.95 | 0.27 |
|  | T3 | 0.38 | 0.44 | 0.44 | 0.21 | 0.99 | 0.39 |
|  | T4 | 0.62 | 0.89 | 0.88 | 0.82 | 0.47 | 0.57 |
|  | T5 | 0.78 | 0.58 | 0.51 | 0.50 | 0.58 | 0.31 |
|  | T6 | 0.94 | 0.59 | 0.36 | 0.46 | 0.71 | 0.09 |
|  | **C3** | 0.68 | 0.80 | 0.30 | 0.79 | 0.27 | **0.01** |
|  | F3 | 0.32 | 0.52 | 0.69 | 0.24 | 0.54 | 0.27 |
|  | F7 | 0.27 | 0.48 | 0.42 | 0.56 | 0.63 | 0.12 |
|  | Fz | 0.39 | 0.47 | 0.37 | 0.32 | 0.81 | 0.89 |
| **m=3, L=1** | O1 | 0.49 | 0.39 | 0.09 | 0.98 | 0.85 | 0.95 |
|  | O2 | 0.18 | 0.98 | 0.66 | 0.80 | 0.62 | 0.27 |
|  | P3 | 0.31 | 0.84 | 0.86 | 0.26 | 0.76 | 0.73 |
|  | P4 | 0.12 | 0.42 | 0.93 | 0.47 | 0.65 | 0.37 |
|  | T3 | 0.49 | 0.33 | 0.51 | 0.15 | 0.40 | 0.28 |
|  | T4 | 0.88 | 0.33 | 0.52 | 0.24 | 0.99 | 0.28 |
|  | T5 | 0.13 | 0.92 | 0.44 | 0.56 | 0.61 | 0.24 |
|  | T6 | 0.59 | 0.89 | 0.48 | 0.73 | 0.43 | 0.40 |
|  | **C3** | 0.67 | 0.83 | 0.28 | 0.57 | 0.34 | **0.02** |
|  | F3 | 0.34 | 0.53 | 0.76 | 0.34 | 0.39 | 0.38 |
|  | F7 | 0.32 | 0.50 | 0.46 | 0.45 | 0.68 | 0.14 |
|  | Fz | 0.40 | 0.48 | 0.31 | 0.51 | 0.93 | 0.96 |
|  | O1 | 0.50 | 0.43 | 0.10 | 0.71 | 0.84 | 0.99 |
| **m=3, L=2** | O2 | 0.18 | 0.98 | 0.59 | 0.75 | 0.86 | 0.25 |
|  | P3 | 0.33 | 0.97 | 0.88 | 0.29 | 0.65 | 0.79 |
|  | P4 | 0.14 | 0.50 | 0.79 | 0.60 | 0.31 | 0.54 |
|  | T3 | 0.49 | 0.32 | 0.51 | 0.16 | 0.75 | 0.32 |
|  | T4 | 0.89 | 0.38 | 0.74 | 0.17 | 0.98 | 0.28 |
|  | T5 | 0.16 | 0.93 | 0.41 | 0.58 | 0.72 | 0.17 |
|  | T6 | 0.54 | 0.97 | 0.56 | 0.84 | 0.29 | 0.33 |

Table II P-values of the correlation analysis of the PEn features using different embedding parameters.

| **Embedding parameters** | **Channel** | **IMF4** | **IMF5** | **IMF6** | **IMF7** | **IMF8** | **IMF9** |
| --- | --- | --- | --- | --- | --- | --- | --- |
| **m=3, L=1** | **C3** | 0.68 | 0.80 | 0.30 | 0.79 | 0.27 | **0.01** |
|  | F3 | 0.32 | 0.52 | 0.69 | 0.24 | 0.54 | 0.27 |
|  | F7 | 0.27 | 0.48 | 0.42 | 0.56 | 0.63 | 0.12 |
|  | Fz | 0.39 | 0.47 | 0.37 | 0.32 | 0.81 | 0.89 |
|  | O1 | 0.49 | 0.39 | 0.09 | 0.98 | 0.85 | 0.95 |
|  | O2 | 0.18 | 0.98 | 0.66 | 0.80 | 0.62 | 0.27 |
|  | P3 | 0.31 | 0.84 | 0.86 | 0.26 | 0.76 | 0.73 |
|  | P4 | 0.12 | 0.42 | 0.93 | 0.47 | 0.65 | 0.37 |
|  | T3 | 0.49 | 0.33 | 0.51 | 0.15 | 0.40 | 0.28 |
|  | T4 | 0.88 | 0.33 | 0.52 | 0.24 | 0.99 | 0.28 |
|  | T5 | 0.13 | 0.92 | 0.44 | 0.56 | 0.61 | 0.24 |
|  | T6 | 0.59 | 0.89 | 0.48 | 0.73 | 0.43 | 0.40 |
| **m=3, L=2** | **C3** | 0.67 | 0.83 | 0.28 | 0.57 | 0.34 | **0.02** |
|  | F3 | 0.34 | 0.53 | 0.76 | 0.34 | 0.39 | 0.38 |
|  | F7 | 0.32 | 0.50 | 0.46 | 0.45 | 0.68 | 0.14 |
|  | Fz | 0.40 | 0.48 | 0.31 | 0.51 | 0.93 | 0.96 |
|  | O1 | 0.50 | 0.43 | 0.10 | 0.71 | 0.84 | 0.99 |
|  | O2 | 0.18 | 0.98 | 0.59 | 0.75 | 0.86 | 0.25 |
|  | P3 | 0.33 | 0.97 | 0.88 | 0.29 | 0.65 | 0.79 |
|  | P4 | 0.14 | 0.50 | 0.79 | 0.60 | 0.31 | 0.54 |
|  | T3 | 0.49 | 0.32 | 0.51 | 0.16 | 0.75 | 0.32 |
|  | T4 | 0.89 | 0.38 | 0.74 | 0.17 | 0.98 | 0.28 |
|  | T5 | 0.16 | 0.93 | 0.41 | 0.58 | 0.72 | 0.17 |
|  | T6 | 0.54 | 0.97 | 0.56 | 0.84 | 0.29 | 0.33 |
